# Measurement of normal retinal vascular pulse wave attenuation using modified photoplethysmography

**DOI:** 10.1371/journal.pone.0232523

**Published:** 2020-05-07

**Authors:** Anmar Abdul-Rahman, William Morgan, Dao-Yi Yu

**Affiliations:** 1 Department of Ophthalmology, Counties Manukau DHB, Auckland, New Zealand; 2 Centre for Ophthalmology and Visual Science, The University of Western Australia, Perth, Australia; 3 Lions Eye Institute, University of WA, Perth, Australia; The University of Melbourne, AUSTRALIA

## Abstract

Pulse wave attenuation characteristics reflect compliance and resistance properties of the vessel wall as well as initial pulse generation factors. Recently, it has become possible to measure and map the retinal vessel wall pulse wave amplitudes. Predictable pulse wave amplitude distribution may allow inferences to be made concerning vascular compliance and resistance. Twenty-eight eyes from sixteen subjects (8 male and 8 female) were examined using modified retinal photoplethysmography with simultaneous ophthalmodynamometry. This allowed the assessment of vessel wall pulsation amplitudes under a dynamic range of intraocular pressures. Pulse amplitudes were calculated using harmonic regression analysis. The pulse wave attenuation was measured under different ranges of ophthalmodynamometric force (ODF) as a function of distance along the vessel (V_Dist_), which in turn was calculated in disc diameters (DD) from the center of the optic disc. A linear mixed-effects model with randomized slopes and intercepts was used to estimate the correlations between the logarithmically transformed harmonic regression wave amplitude (HRW_a_) and the Fourier trigonometric coefficients with the predictors (V_Dist_ and ODF). The retinal venous harmonic regression wave attenuation (coefficient value±standard error) -0.40±0.065/DD, (p-value < 0.00001, 95% confidence interval (CI) -0.53 to -0.27), which was approximately twice that of the arterial -0.17±0.048/DD, (p-value < 0.0004, 95% CI = -0.27 to -0.08). There was a positive correlation between attenuation of the harmonic regression wave and ophthalmodynamometric force in both vascular systems. The attenuation of all but the sine coefficient of the second Fourier harmonic (b_n2_) achieved statistical significance in the correlation with V_Dist_. The cosine coefficient of the first Fourier harmonic a_n1_ was the only coefficient to achieve statistical significance in the correlation with the predictors V_Dist_ and ODF in both vascular systems. The a_n1_ coefficient value in the correlation with V_Dist_ was -3.79±0.78 and -1.269±0.37 (*p* < 0.0006), while this coefficient value in the correlation with ODF was 0.026±0.0099 and 0.009±0.04 (*p* < 0.01) in both the retinal veins and arteries respectively. The predictable attenuation characteristics in normal subjects suggest that this technique may allow the non-invasive quantification of retinal vascular compliance and other hemodynamic parameters.

## Introduction

The vascular pulse wave is a mechanical pressure wave propagating in the wall of the blood vessel at a velocity different to that of blood flow but physiologically coupled to flow through the radially directed transmural pressure and a shear stress force, which is oriented in a parallel direction to the blood vessel wall [1, 2]. The retinal venous pulse is a useful parameter in the management of ophthalmic and systemic disorders such as glaucoma [3–5], increased intracranial pressure [6, 7], thyroid orbitopathy [8], retinal vein occlusion [9–12] and diabetic retinopathy [13, 14]. The physiologic mechanisms of the retinal venous pulse are poorly understood; being generated by the heart and transmitted via the cerebrospinal fluid space, it propagates in the wall of the central retinal vein in a retrograde direction to blood flow, entering the eye through the optic cup, where it attenuates (decays in amplitude) rapidly [13, 15]. The arterial pulse is the result of a wave of vascular wall distention, initiated by the impact of the stroke volume ejected into a closed system [16]. Amplification of the arterial pulse wave contour at points downstream is attributable to vessel wall taper. Further modification of the pulse wave contour occurs as a consequence of pulse wave reflection from junctions and discontinuities in the arterial tree [17]. In addition, there may be a frequency-dependent attenuation of the vascular pulse wave amplitude and propagation velocity by the mural viscoelastic elements. Vascular compliance is the main factor determining pulse wave attenuation through a positively correlated relationship (i.e. the higher the compliance, the higher the pulse wave attenuation) [18–21]. Compliance varies with vessel wall viscoelasticity and tension in the wall of the blood vessel per unit length (T); expressed through Laplace’s law [2, 22–25] as a product of the transmural pressure (p_tm_) and the radius (r) of the blood vessel (*T* = *p*_*tm*_ ⋅ *r*).

To date the Dynamic Vessel Analyzer is the only commercially available method to measure the retinal vascular pulse wave properties synchronized with the cardiac cycle, which theoretically can measure retinal vascular pulse wave attenuation [26–28]. In principle it assesses retinal vessel diameter by analyzing the brightness profile of the vessel using video sequences obtained with a conventional fundus camera. To achieve an optimum contrast for vessel visualization, a green filter is inserted into the illumination pathway of the fundus camera. It is available in two versions: the retinal vessel analyzer (RVA), which is designed for research purposes and the dynamic vessel analyzer (DVA), intended for clinical use and includes a capacity to provide flicker light stimulation as a provocative test. Its adaptive algorithm, based on variation in brightness, compensates for reflections during measurement and, additionally, the instrument can automatically compensate for artifactual movements. Images deemed to be of poor quality are automatically rejected from the analysis. As with any optical device measurement accuracy is degraded with optical opacity and in patients with limited fixation. However, the main limitations include the inability to perform measurements where vessels are in close proximity or when the vessel cross-sectional diameter is <90μm [29]. Retinal vessel diameters are reported in relative units of measurement as determined by the Gullstrand eye, which can result in scaling errors if the eye has a refractive error or its dimensions vary from that of a schematic eye. Although provocative tests are possible with the DVA, there is limited data on the reproducibility of flicker responses [30].

Chen et al. described an imaging system consisting of a standard Zeiss 30° fundus camera with monochromatic red-free photographs, synchronized with the cardiac cycle using an ECG monitor connected to the camera through a time delay switch, which was set to release the camera shutter at a chosen period within 1/8th of the cardiac cycle. The changes in retinal vascular diameter were measured manually at consecutive intervals. They concluded that both retinal arterial and venous diameters underwent a change concomitant with the events of the cardiac cycle. The arterial diameter reached a maximum in mid systole, which is just after the period of peak aortic pressure, whereas the increase in intraocular pressure during systole, lead to a reduction in the retinal venous distending pressure and consequently resulted in a passive reduction of the venous diameter [31]. Limitations of this method include the manual technique used to measure vessel diameters at the same site in different image sequences and, in addition, only a single point in each vessel was examined [31]. Kumar et al. modified this technique using an image analysis method coined “Vesselness Mapping of Retinal Image Sequence”, which detected and filtered vessel boundaries enhanced by the scale-space analysis of the eigenvectors of the image Hessian matrix. This technique was used to examine a longer segment of the vessel and, consequently, several pulsatile features other than spontaneous venous pulsations were detected including serpentine movements, vessel displacement and mechanical coupling. However, identification of the pulsatile segment was required before measurement [32]. Moret et al. used near-infrared slit-lamp ophthalmoscopy (HRA-OCT Spectralis) and applied principal component analysis to evaluate the image sequence. They also reported the detection of various forms of vascular pulsation including serpentine movement of the principal arteries, spontaneous venous pulsation, pulsatile movement of the entire optic nerve head, vessel displacement and mechanical coupling. Although the near-infrared illuminating light beam precluded the need for mydriasis, the advantage of this technique was offset by a low frame rate of 9 frames/second [33]. Recently swept-source optical coherence tomography was used to measure the retinal vascular axial expansion during the cardiac cycle, by analyzing the increase of the retinal thickness caused by the reversible expansion of the vessels. Using this method, Spahr et al. reported the detection of a separate retinal arterial and venous pulse wave. They detected the time delay between the two waves and estimated the pulse wave velocity in the retinal arteries to be 620±50 mm/s [34]. This is about 1,500 times faster than that measured using the RVA [27]. However, the technique failed to measure the retinal vein pulse wave velocity [34].

Photoplethysmography (PPG) is a non-invasive optical technique used to detect blood volume changes in tissue microvascular networks. The basic form of PPG technology consists of a light source to illuminate the tissue, and a photodetector to measure the variations in the intensity of transmitted or reflected light associated with changes in perfusion in the tissue volume of interest [35]. PPG technology has been used in a wide range of commercially available medical devices including the measurement of oxygen saturation [36], blood pressure [37–39], cardiac output [40, 41], the assessment of autonomic function [42, 43] and the detection of peripheral vascular disease [44–46]. The principle of operation is described by the Beer-Lambert Law, which describes the relationship between a uniform medium containing an absorbing substance and light attenuation [36]. We modified this system to objectively measure vessel pulsation amplitude from sectors of retinal images centered on the optic disc and from optic disc surface vessels, using retinal video recordings spanning three cardiac cycles [47, 48]. Modified photo-plethysmography (mPPG) allows the estimation of the attenuation of the retinal vascular pulse wave in both the retinal arteries and veins, within one disc diameter of the optic disc. In addition to being non-invasive and low cost, it offers further advantages by continuously measuring both the arterial and venous pulsation amplitudes separately. The transparent ocular refractive media allows mapping and display of the spatial distribution of the mPPG data in two dimensions. Pulse oximetry, recorded simultaneously with the optic nerve video recording, provides the necessary audio feedback, which is then used for the extracted rasterized image frames to be timed with the cardiac cycle. Time-series analysis is then used to quantify the retinal vascular pulse wave parameters. Additionally, ophthalmodynamometry adds a dynamic aspect, by altering the retinal vascular pulse amplitude over a range of intraocular pressures [47–49]. The purpose of the study was to estimate the retinal vascular attenuation non-invasively and provide the response of the attenuation characteristics to changes with induced intraocular pressure in a group of normal subjects.

## Materials and methods

Healthy adult research participants were recruited from the medical student body and relatives of patients treated at the Lions Eye Institute. Written consent was obtained from each of the participants. Study approval was obtained from the University of Western Australia Human Ethics Committee adhering to the tenets of the Declaration of Helsinki. From the 32 eyes of 16 subjects, 28 were included in the analysis as four eyes were excluded due to poor image quality. Participants were required to have clear ocular media and a normal retina and optic nerve. Exclusion of a functional retinal or optic nerve pathology was determined by Humphrey standard automated perimetry or Frequency Doubling Perimetry (Humphrey Zeiss, Dublin, Ca). Subjects were not excluded based on smoking history or systemic hypertension. All subjects rested for half an hour, they had visual field testing, then dilated after another half hour. They were neither given any caffeine nor was the blood pressure measured during this period.

### Ophthalmodynamometry technique

The Meditron ophthalmodynamometer (Meditron GmbH, Poststrasse, Völklingen, Germany) consists of a three-mirror Goldmann contact lens fitted at the observer end with a ring-shaped force transducer. The force transducer consists of five parts: (1) a metallic holding grip with (2) a metallic cover on the outer surface acting to compress flexible structures in the interior part of the holding grip; (3) the flexible trabeculae composed of copper-beryllium and acting as the main pressure measuring unit; (4) a metallic structure on which the trabeculae rest and (5) the plastic ring which is in contact with the contact lens and which is usually taken to hold the Goldmann contact lens [50]. The sensor ring is connected to a liquid crystal display monitor, on which the force continuously measured by the sensor ring is displayed. A foot pedal is connected to the display monitor, which is used to communicate the ophthalmodynamometric force to the display. The examination commenced with the calibration step, the observer end of the Goldman contact lens was rested on a flat surface and the device was activated. After initialization, an audio signal indicated a successful calibration. Baseline intraocular pressure was measured, the subjects’ pupils were dilated with 1% Tropicamide. The Goldmann contact lens of the ophthalmodynamometer was placed on the topically anesthetized corneal surface. Contact gel was applied to the lens to ensure optical interface continuity with the cornea. The examination was performed by applying gradual increments in pressure onto the contact lens up to a constant force at the observers’ discretion; the aim is to induce visible pulsations in the central retinal vein and maintain a constant force. The force measured by the sensor ring surrounding the Goldmann contact lens was then read by pressing on the foot pedal; the displayed reading was noted by a second observer. This was the endpoint of each test run. The optic nerve head was continuously imaged bio-microscopically during the examination through a Meditron Ophthalmodynamometer. The examination was repeated between 8 and 10 times for each subject to attain a range of ODF values for each eye. Videos showing excessive motion artifact, reflection from the optical media or decentration of the optic nerve in the image sequence for less than three consecutive cardiac cycles were rejected from the analysis. The ophthalmodynamometric force (ODF), displayed as Meditron units (mu), was converted to grams of force, where 1 mu = 3.33 grams of force and induced IOP = 0.89xODF+Baseline IOP in mmHg [51].

**Fig 1:**
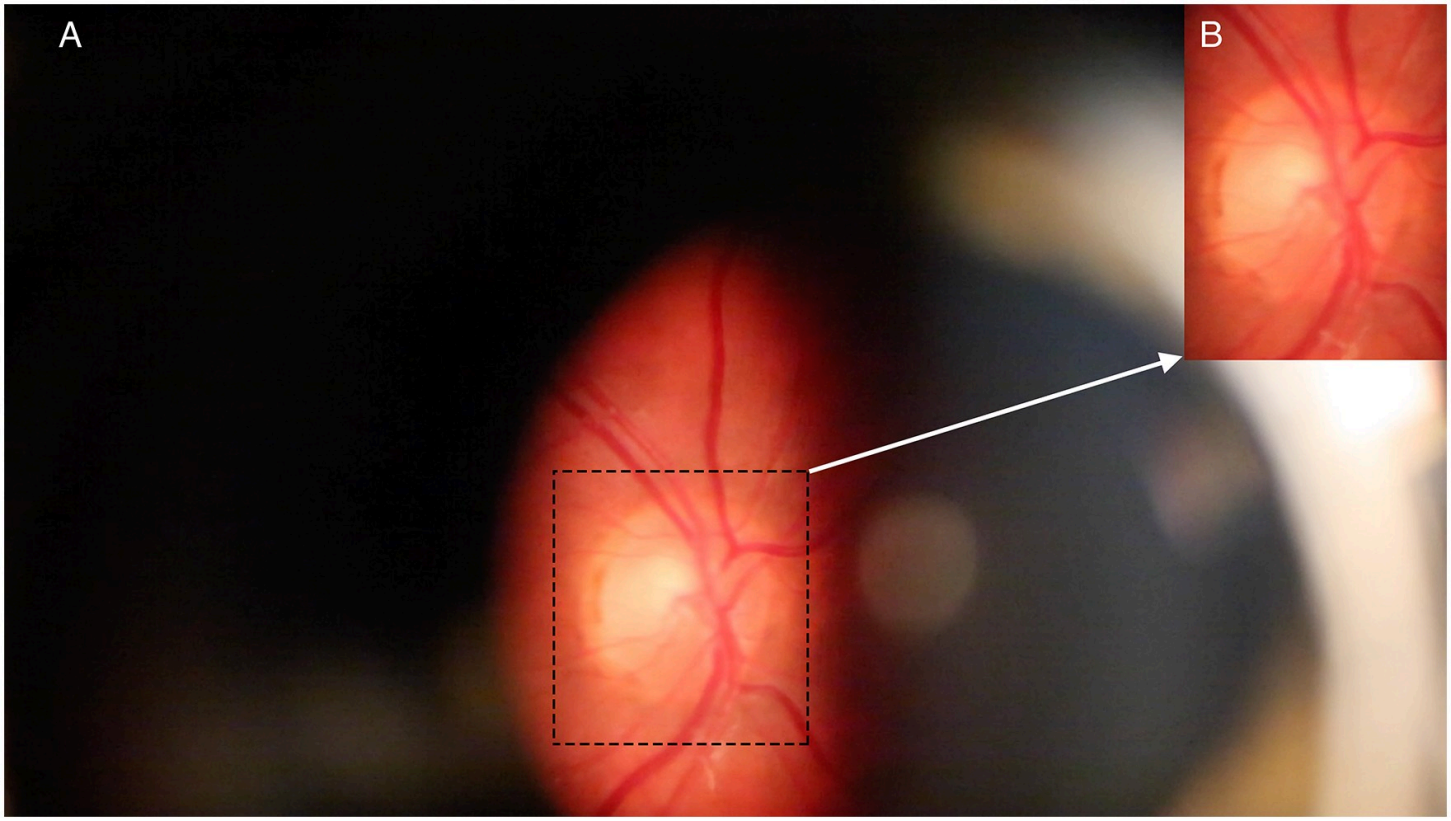
Example of the image zone of analysis. A) Full frame image as viewed through the Meditron ophthalmodynamometer. B) Inset showing the region of interest after cropping the image.

An imaging slit-lamp (Carl Zeiss, Germany) with a digital camera (Canon 5D Mark III, Japan) was used for retinal imaging. Several sequences of at least three cardiac cycles in length were taken (each at a rate of 25 frames/second). When possible, recordings were taken from both eyes. A pulse oximeter (Nellcor N65, Covidien, Mansfield, MA) was applied to the right index finger; the audio signal from the pulse oximeter, captured with the video sequence of the optic nerve, allowed synchronization of the retinal vascular pulse with the cardiac cycle. The timing of the cardiac cycle, was generated from the audio signal from the subject’s pulse oximetry recorded on the audio trace of the video segment, enabled the mathematical analysis of the periodic component from green channel transmittance. A single high quality three cardiac cycle length video recording was extracted from each raw recording session. Individual video frames were imported as separate images in (.tif) format into Photoshop (CS6). The portion of the images containing the optic disc and surrounding retina was cropped to exclude parts of the image at the boundary of the slit lamp illumination beam (Fig 1).

### Principles of image analysis

All images from three cardiac cycles were analyzed in R statistical package [52]. Image analysis and segmentation are detailed in our previous work [13, 53]. The principle of the optical path length and the relationship to hemoglobin concentration is described by the Beer-Lambert Law (Eq 1).

$$\begin{array}{c} A=\varepsilon (\lambda )\cdot c\cdot d=-lnT \end{array}$$ (1)

ε(λ) = Extinction coefficient or absorptivity of the medium for a specific wavelength (L/mmol/cm)

c = Concentration of light absorbing material which is assumed to be a constant (mmol/L)

d = Optical path length through the medium (cm)

T = Transmittance of a material sample

The isosbestic point, defined as the wavelength where light absorbance for both oxy- and deoxyhemoglobin, is approximately 550 nm (green channel), therefore the green color channel from each image layer was extracted [54, 55]. To mimic the physiology of the human eye, the Bayer filter mosaic used on video camera sensors favors the green channel due to the filter pattern arrangement of 50% green, 25% red and 25% blue, [56] The fact that there are twice as many green filters results in the green channel being the least noisy of the three color channels. There is a variation of hemoglobin content in a pulsating vessel, resulting in a fluctuating retinal light transmittance during the cardiac cycle [13]. For Eq 1 the uniform absorbance (A), is proportional to the negative logarithm of transmittance (T), this is approximated in each cardiac cycle using the green color channel and by assuming an extinction coefficient ε(λ) of 12 L/mmol/cm at 550 nm for both oxy and deoxyhemoglobin and a hemoglobin concentration (c) of 150g/l [13]. To convert from molar extinction coefficient to absorbance a molar density of 64,500 g/mol was used and the result divided by 2 as a reflectance method assumed that light was reflected through the same vessel twice, doubling the optical path length.

$$\begin{array}{c} A=\frac{\epsilon (\lambda )\cdot c\cdot d}{64,500}\cdot \frac{1}{2} \end{array}$$ (2)

If the various assumptions are correct and absorption was limited to 550 nm then the calculated optical path length would be in microns. This value has not been validated empirically and the mathematical derivation is a marked simplification of the complexities of matching between hemoglobin transmittance, reflectance, scatter and the charge-coupled device green channel sensitivity profile. Absolute vessel dimensions would also depend upon knowledge of the incident light upon the retina to calculate absolute transmission. We substitute this information by calculating the difference in these values and their rates of change across the cardiac cycle, which also gives informative results [13].

### Data processing

Image processing was done in Adobe Photoshop CS6. The video-recording was performed through an ophthalmodynamometer lens system. Individual image frames were extracted from each video sequence and saved as Tagged Image File Format (TIFF) files. Each of these images was cropped to an array of pixels with coordinates x, the pixel at location x has an associated intensity triple I(x) = (R(x), G(x), B(x)), where R, G, and B respectively describe the red, green and blue levels [57]. The intensity triples were extracted using the R package jpeg or tiff [58], with RGB values converted to the standard [0, 255] scale. Following this procedure, the data for any given video are represented as a sequence I_1_(x),I_2_(x)….I_M_(x), where M is the total number of frames, typically M = 70 for each video clip to include three cardiac cycles. Each sequence of images was rasterized and aligned. Image segmentation was performed manually, by creating a set of four vessel templates to isolate portions of each image corresponding to two parts (A and B) of the lower retinal vein, the upper vein, and the artery. To account for both noise and the central vessel reflex, pixel RGB intensity appearing as almost pure white [I = (255, 255, 255)) or black (I = (0, 0, 0))], with RGB mean intensity values within 1% of either of these extremes, were excluded from the subsequent analysis. (This affected less than 0.00001% of the pixels across our video data). The mean color intensity over a template reflects the aggregate blood volume in the corresponding section of the retina; for any given template (T) the information was summarized after excluding pixels with extreme values as outlined above. The information was summarized by computing means for each of the RGB channels and plotting the time series of these values: [R(x): x∈T], [G(x): x∈T] and [B(x): x∈T] [47]. Data from the green channel was extracted as outlined above. A darker image was registered as having a higher intensity and therefore a thicker blood column with more hemoglobin content. Therefore, the change in the mean image intensity of the green channel, is proportional to the change in blood volume. An array of values across the entire optic disk and peripapillary retina from either 2x2 or 5x5 pixel clusters was then used to generate false-color maps based upon the values of either the amplitudes, slopes or timing information [13].

### Mathematical model

A time series is any metric measured over regular time intervals [59]. In our model each data point represented by the mean of the green channel intensity (y(t)) at time (t) is measured as a fraction of the cardiac cycle, rather than in seconds. Eq 3 represents the nominal time for frame (i) in cycle (c, where c = 1-3).

$$\begin{array}{c} t_{i}=\frac{i}{n_{c}}+c-1 \end{array}$$ (3)

where n_c_ = number of frames in the cth cycle.

The components of the series were expressed as a sum of the trend or regular term (f(t)) and the stationary error, irregular or residual term (ε_t_) with a zero mean (Eq 4).

$$\begin{array}{c} y(t)=f(t)+\epsilon_{t} \end{array}$$ (4)

The trend component was decomposed into periodic f(t)_p_ and non-periodic components f(t)_np_, the latter is to account for changes in intensity due to subject movement artifact (Eq 5).

$$\begin{array}{c} f(t)={f(t)}_{p}+{f(t)}_{np} \end{array}$$ (5)

The periodic trend component was modeled as a Fourier series expansion (Eq 6), this approach is used in the field of computational fluid dynamics of oscillating flow [60, 61].

$$\begin{array}{c} F\left({f(t)}_{p}\right)=a_{0}+\sum_{n=1}^{\infty }a_{n}\cdot cos\left(n\pi t\right)+b_{n}\cdot sin\left(n\pi t\right) \end{array}$$ (6)

a_0_ = Coefficient representing the mean of f(t)_p_.

a_n_ = Coefficient of the cosine function of f(t)_p_.

b_n_ = Coefficient of the sine function of f(t)_p_.

n = Integer 0,1,2… etc. representing the harmonic component.

Higher harmonic frequency model comparisons were conducted using the Akaike Information Criterion (AIC). In most eyes AIC preferred models with first and second-order frequencies (Eq 6), therefore the final analysis was limited to the first and second harmonics [47].

The non-periodic component of the trend f(t)_np_ was modeled using a linear spline with knots at times t = 1 and t = 2 (Eq 7). Knot frequency was based on the observation that the duration of most artifactual movements is at least one second, which in turn is approximately equal to one cardiac cycle.

$$\begin{array}{c} {f(t)}_{np}=b_{0}+b_{1}t+b_{2}{\left(t-1\right)}_{+}+b_{3}{\left(t-2\right)}_{+} \end{array}$$ (7)

where the subscript + indicates truncation below at zero, so that z_+_ = z for z>0 and z_+_ = 0 for z≤0 [47]. The error component of the series described by Eq 3 was modeled using a first- order autoregressive process (i.e. the value of the point in the series is weighted by a value of a proceeding datapoint, separated by one lag in the time series), so as to account for residual serial dependence in the data.

$$\begin{array}{c} e_{\text{t}}=\rho \epsilon_{\text{t}-1}+u_{\text{t}} \end{array}$$ (8)

where u_t_ is white noise and *ρ* is the autoregressive parameter estimated by restricted maximum likelihood (REML).

**Fig 2:**
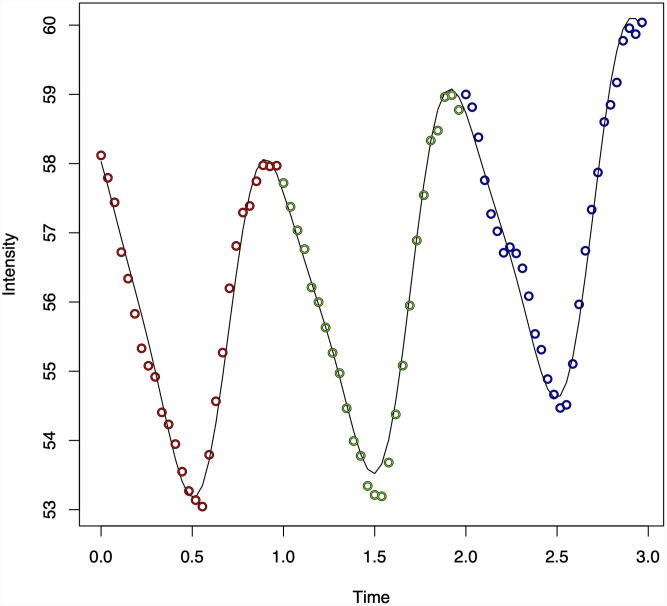
Harmonic regression model fit in a single case. The periodic nature of the data and harmonic regression model fit from the green channel from three cardiac cycles is demonstrated in the retinal vein from a single case. The changes in the harmonic regression waveform with changes in ODF are detailed in Figs 9 to 11.

**Fig 3:**
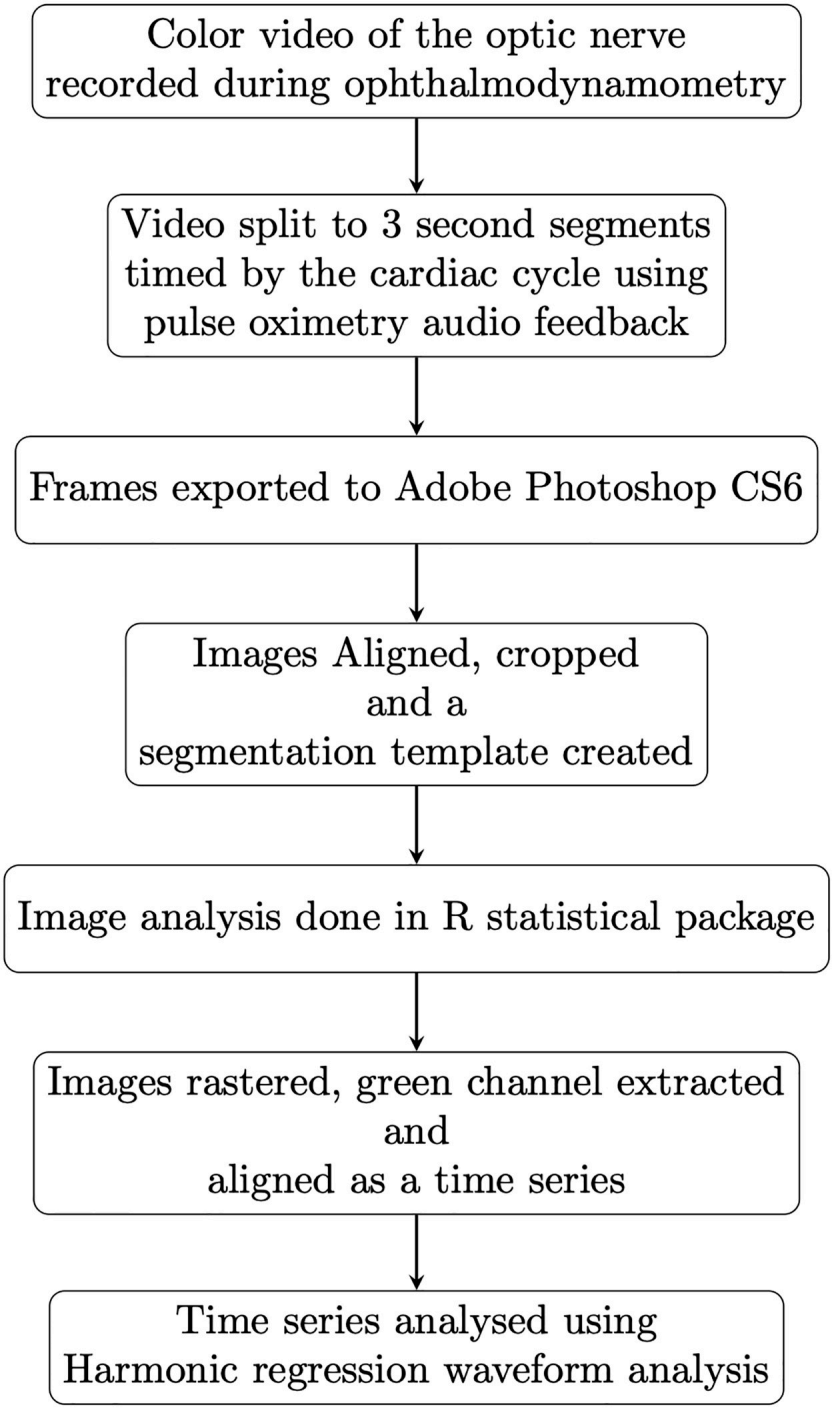
Summary workflow. Schematic of the technical workflow in the data acquisition and image processing.

The periodic nature of the data and model fit from the green channel from three cardiac cycles is demonstrated for a single case in (Fig 2), the model fit for the three channels is further detailed in our earlier work [47]. A summary of the workflow is demonstrated in (Fig 3).

### Statistical analysis

The harmonic regression model is a time series (Eq 3), with both a harmonic trigonometric series and autoregressive error terms. The amplitude of the composite (combined first and second harmonic waveforms) was termed the harmonic regression wave amplitude (HRW_a_) [47], which was modeled for the arteries and veins separately. As the distribution of the HRW_a_ was non-normal, the median was used to measure central tendency; range and interquartile range (IQR) were used to measure data dispersion. The Kruskal-Wallis test was used to assess the statistical significance between the median HRW_a_ of both vascular systems. HRW_a_ was normalized using logarithmic transformation, which is a recommended transformation for data skewed to the right [62]. In addition, Pearson’s criteria for normality were used to confirm the appropriate transformation, these values were HRW_a-arterial_ = 1.32 and HRW_a-venous_ = 3.77, where a value closer to zero confirms the suitability of the methodology. The biological principle underpinning this transformation is the fact that viscoelastic stress-strain curves in blood vessel walls are curvilinear [19], which is approximated with a logarithmic function within the physiologic range. Analysis of variance (ANOVA) was used to estimate the F-statistic and p-value of the HRW_a_ for the difference in attenuation between vessel type. As the spatial profile of the pulse wave amplitude distribution is influenced by the point of maximum pulsation within a vessel, a linear mixed random intercept-random slope model with interaction effects was used to analyze the correlations of both the HRW_a_ and individual trigonometric harmonic coefficients [cosine (a_n_), sine (b_n_)] of the first and second harmonics [cosine (a_n1,2_) and sine (b_n1,2_)] with the predictors, both the distance along the vessel (V_dist_) and ophthalmodynamometric force (ODF). A p-value of <0.05 was considered statistically significant for all analyses [63, 64].

To account for the variances of each of the random factors and the residual variance in the structure of the linear mixed effect model, fit statistics was assessed using conditional R^2^, which describes the proportion of variance explained by all factors in the model including the predictors V_dist_ and ODF and random factors (subject, age, gender, laterality “right/left”, hemiretinal location “superior/inferior”), whereas marginal R^2^ describes the proportion of variance explained by the predictors V_dist_ and ODF alone [65]. Multivariate coefficient effect size was calculated for the terms in the interaction model using standardized weighted (β_σ_) coefficients [66]. In all regression models the coefficients were reported ±standard error. The 95% confidence intervals were calculated for all regression coefficients.

## Results

**Fig 4:**
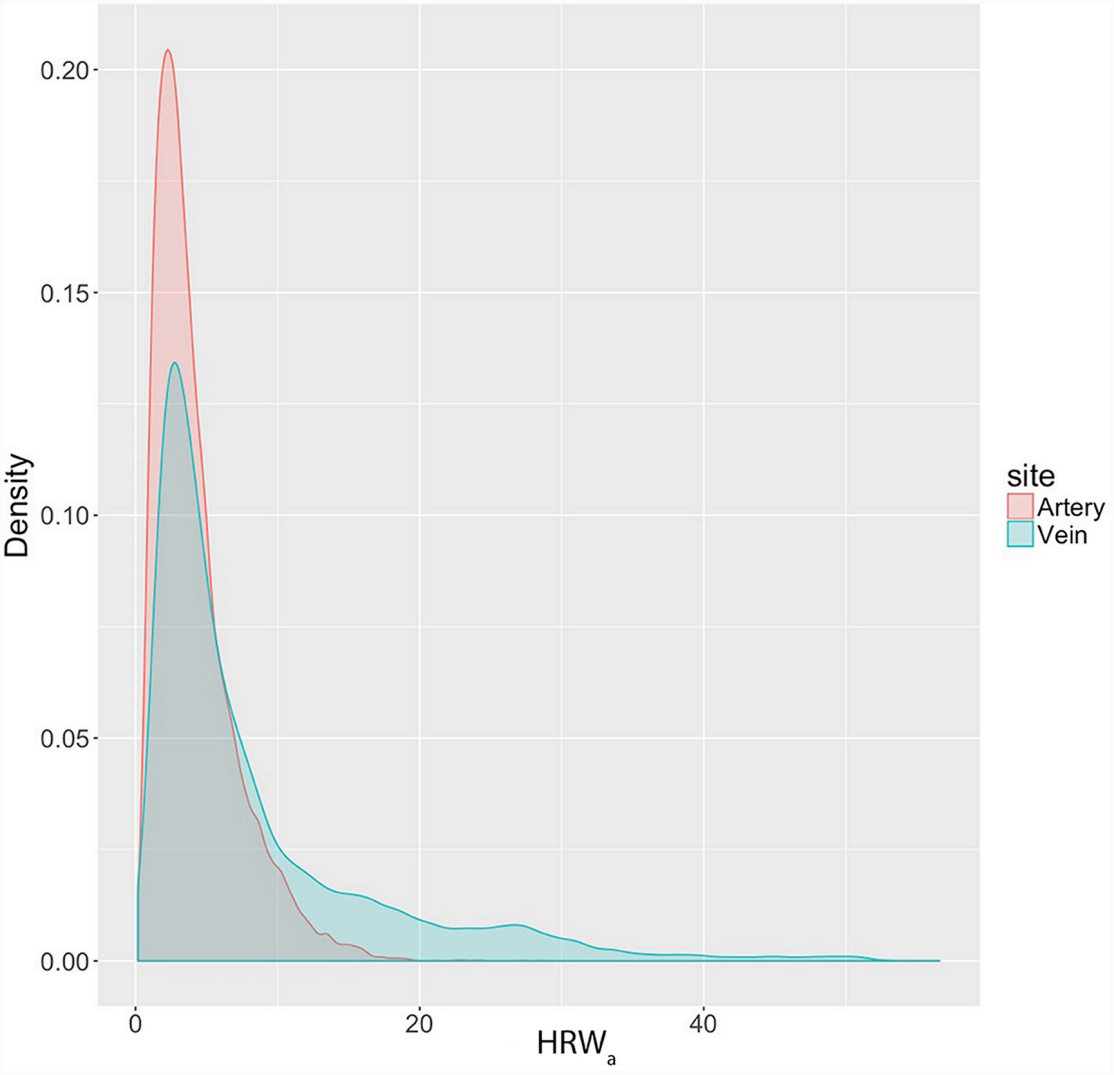
Density plot of the harmonic regression wave amplitude (HRW_a_) distribution in the retinal vascular system. The distribution of the HRW_a_ is non-normal, demonstrating a right skew in both retinal vascular systems. A larger skew in the HRW_a_ distribution is demonstrated from values from the retinal veins compared to the retinal arteries.

From a sample of sixteen subjects (8 male and 8 female), a total of 28 eyes were examined. The median age was 48 years (range = 22-69 years). The distribution of the HRW_a_ was skewed to the right (skewness = 1.58 and 2.03, kurtosis = 3.35 and 4.44) for both the retinal arteries and veins respectively (Fig 4). The median HRW_a_ in the venous system was 5.11 (range = 0.16–56.6, IQR = 7.64), compared to the arterial system 3.36 (range = 0.20–28.54, IQR = 3.28), the difference between the median values was statistically significant (p<0.00001).

**Fig 5:**
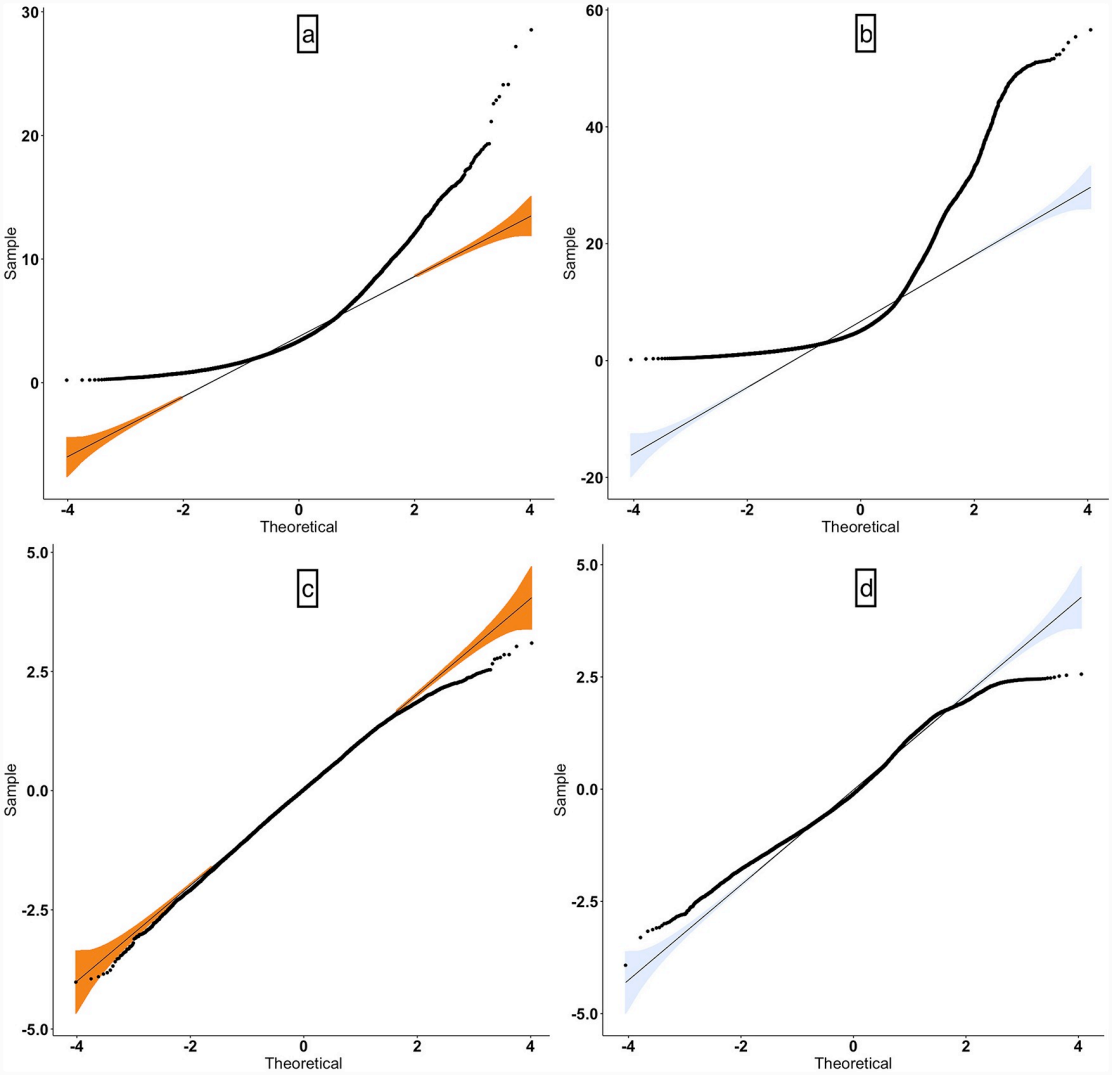
Normal quantile-quantile plot (q-q plot). A normal q-q plot is a graph of the sets of quantiles of the dataset plotted against the quantiles of a normal distribution. The upper row (a,b) represents non-transformed and the right (c,d) logarithmically transformed harmonic regression wave amplitude (HRW_a_). There is an improved approximation to a normal distribution after transformation.

Due to the non-normal distribution of the HRW_a_ in the venous and arterial systems, logarithmic transformation was performed (skewness logHRW_a-arterial_ = -0.21, logHRW_a-venous_ = 0.20), normal quantile-quantile plots (q-q plots) in Fig 5 demonstrated a favorable approximation of the residuals to a normal distribution. The mean logarithmically transformed ±standard error of the mean (SEM) HRW_a_ was greater in the venous system 0.84±0.034 (95% confidence interval (CI) = 0.77–0.92), than the arterial system 0.65±0.025 (95% CI = 0.60–0.70).

**Fig 6:**
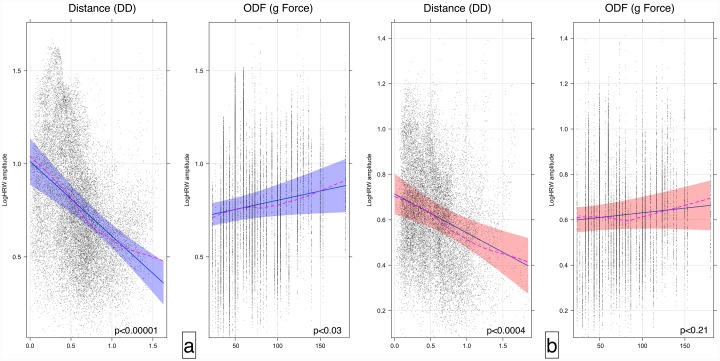
Effect plots of the retinal vascular system. Effect plot of the retinal veins (a) and retinal arteries (b) highlighting the correlation between the vascular pulse wave log harmonic regression waveform amplitude (HRW_a_), distance along the vessel from the center of the optic disc (V_Dist_) in disc diameter and ophthalmodynamometric force (ODF) in grams force (g). Noted is the attenuation of the HRW_a_ with distance compared to the amplification by ODF. Demonstrated are the 95% confidence intervals for the slope and intercept of the regression line. The dashed line is the loess smoothed regression curve, superposition of both the curve and the regression line indicates a favorable model fit.

The retinal vascular pulse attenuation was estimated by calculating the regression coefficients correlating log HRW_a_ and V_dist_. A higher attenuation was seen in the venous system -0.40±0.065/DD, (p-value < 0.00001, 95% confidence interval -0.53 to -0.27) as opposed to the arterial system -0.17±0.048/DD, (p-value < 0.0004, 95% CI = -0.27 to -0.08). The difference in the attenuation coefficients was compared using ANOVA and was found to be statistically significant (F-statistic = 10751.3, p<0.0001). In contrast to the attenuation of HRW_a_ with distance along the vessel (V_Dist_), there was an increase of the log HRW_a_ with ODF by 0.00099±0.0005/g force, (p-value<0.03 95% confidence interval 0.000091 to 0.0019) in the venous system. In the arterial system a coefficient of 0.00041±0.0003/g force failed to achieve statistical significance (p-value <0.21, 95% CI = -0.00024 to 0.0011), (Fig 6).

The equations of the regression lines from Fig 6 can be derived from these correlations:

$$\begin{array}{c} logHRW_{a-venous}=1.012-0.40\cdot V_{\text{Dist}} \end{array}$$ (9)

$$\begin{array}{c} logHRW_{a-venous}=0.738+0.00099\cdot ODF \end{array}$$ (10)

$$\begin{array}{c} logHRW_{a-arterial}=0.713-0.17\cdot V_{\text{Dist}} \end{array}$$ (11)

$$\begin{array}{c} logHRW_{a-arterial}=0.599+0.00041\cdot ODF \end{array}$$ (12)

**Fig 7:**
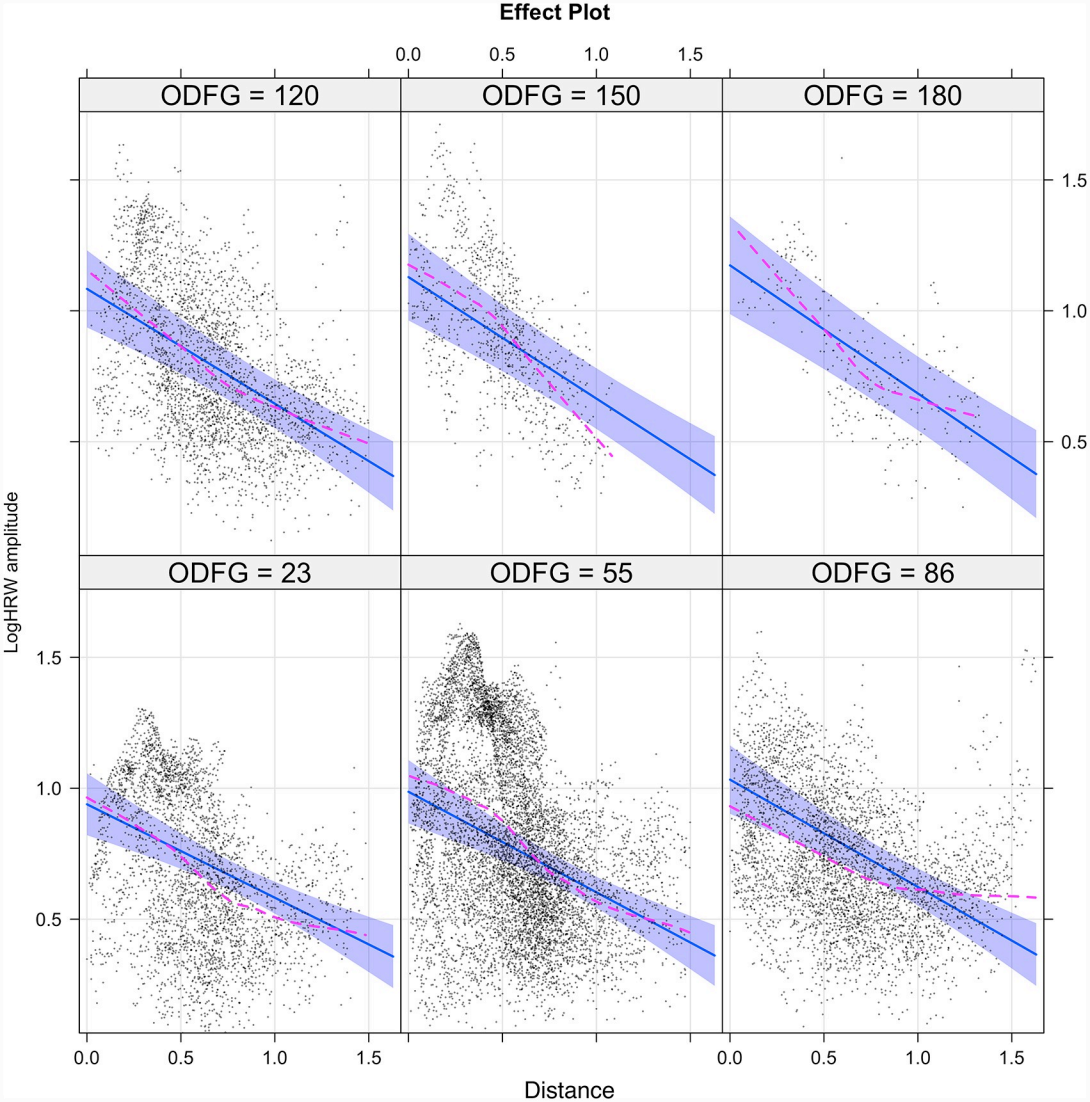
The interaction model of retinal venous pulse log harmonic regression waveform amplitude (logHRW_a_). There was a positive correlation between HRW_a_ attenuation and increasing ODF characterized by steepening of the slope of the regression line from -0.36 at 23g force to -0.49 at 180g force with the maximal change in logHRW_a_ occurring at the center of the optic disc. Demonstrated are the 95% confidence intervals for the slope of the regression line. The sequence commences from the lower left to the upper right. The dashed line is the loess smoothed regression curve, superposition of both the curve and the regression line indicated a favorable model fit.

**Fig 8:**
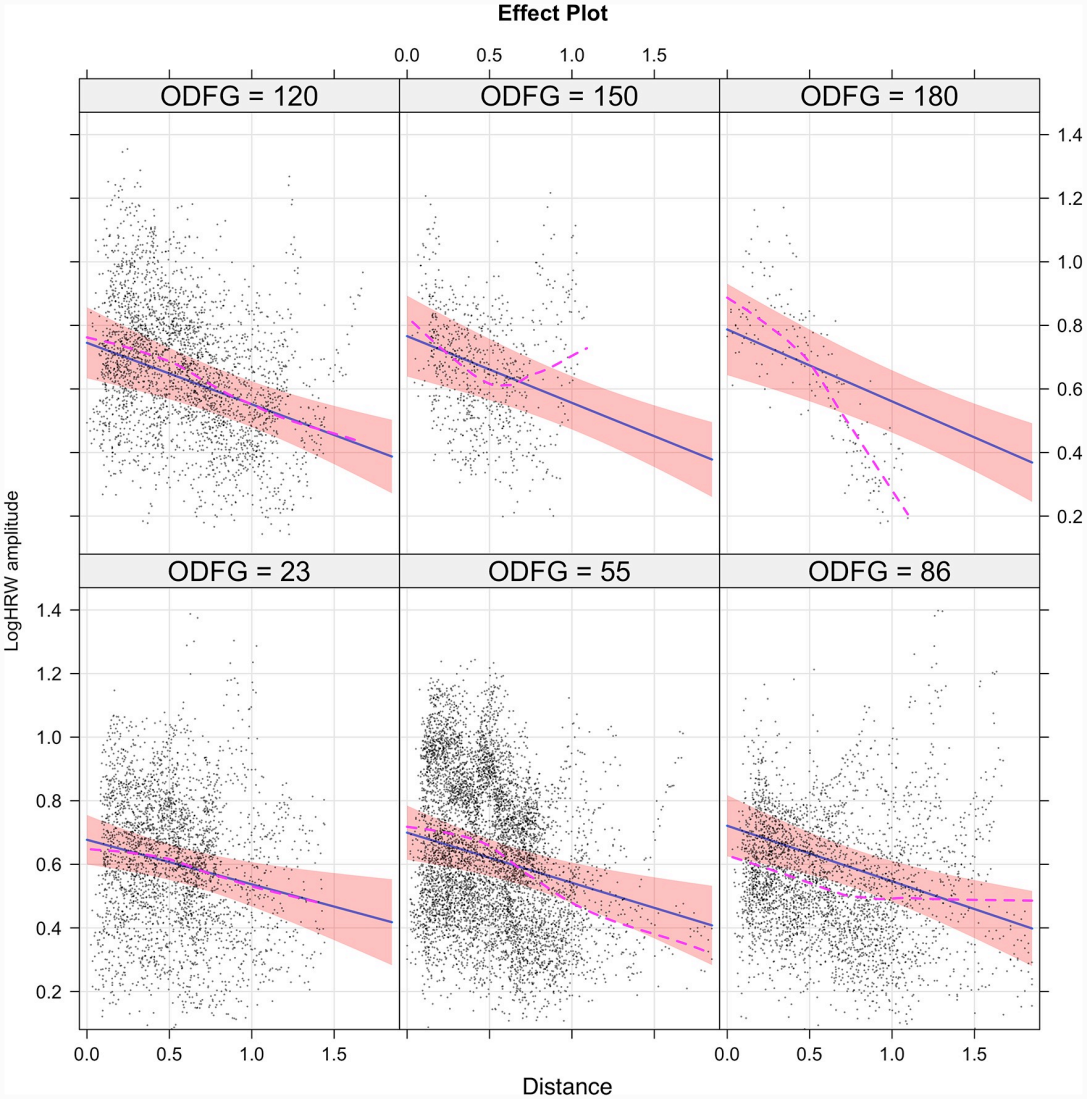
The interaction model of retinal arterial pulse log harmonic regression waveform amplitude (logHRW_a_). There was a positive correlation between HRW_a_ attenuation and increasing ODF characterized by steepening of the slope of the regression line from -0.14 at 23g force to -0.23 at 180g force with the maximal change in log HRW_a_ occurring at the center of the optic disc. Demonstrated are the 95% confidence intervals for the slope of the regression line. The sequence commences from the lower left to the upper right. The dashed line is the loess smoothed regression curve, superposition of both the curve and the regression line indicated a favorable model fit.

The interaction (inter-dependence) of the predictors (V_dist_ and ODF) in estimating the outcome (log HRW_a_) are demonstrated in the Figs 7 and 8 for the retinal veins and arteries respectively. The interaction model yields the following regression equations:

$$\begin{array}{c} logHRW_{a-venous}=0.90-0.34\cdot V_{\text{Dist}}+0.0015\cdot ODF-0.00084\cdot V_{\text{Dist}}\cdot ODF \end{array}$$ (13)

$$\begin{array}{c} logHRW_{a-arterial}=0.66-0.13\cdot V_{\text{Dist}}+0.0007\cdot ODF-0.0005\cdot V_{\text{Dist}}\cdot ODF \end{array}$$ (14)

The interaction model coefficients, p-values and standardized (β_σ_) coefficients are summarized in Table 1, from which the following conclusions could be drawn: 1) although more than 60% of the variance was explained by the combined predictors and the random factors of the model (conditional R^2^), the tested predictors alone accounted for less than 21% of the variance (marginal R^2^). 2) There was an attenuation of the HRW_a_ with V_Dist_ and amplification with ODF as evidenced by a negative coefficient for distance (C_VD_) and a positive coefficient for ODF (C_ODF_), respectively, of which both coefficients were higher in the veins compared to the arteries. The standardized β_σ_ coefficients (β_σVD_ and β_σODF_) seen in Table 1 demonstrated this relationship. 3) From the interaction plots in Figs 7 and 8 it can be noted that the slope of the attenuation with V_Dist_ is more negative with increasing ODF. This could be quantified by the negative interaction term (C_VD*ODF_). In both vascular systems this change is maximal at the center of the optic disc and reduces towards the retinal periphery.

**Table 1:** Linear mixed effects model with interaction of the predictors.

Interaction Model Parameters		Retinal Vessels			
		Veins	95%CI	Arteries	95%CI
Model Variance	Conditional R^2^	0.66		0.63	
	Marginal R^2^	0.21		0.06	
Model Coefficient±SEM	I_0_	0.90±0.06***	0.78 to 1.02	0.66±0.04***	0.58 to 0.74
	C_VD_	-0.34±0.07***	-0.47 to -0.21	-0.13±0.05*	-0.24 to -0.03
	C_ODF_	0.0015±0.0005**	0.0005 to 0.002	0.0007±0.001*	0.0001 to 0.00003
	C_VD*ODF_	-0.00084±0.0002***	-0.001 to-0.0005	-0.0005±0.0001***	-0.002 to -0.0008
Standardized (β_σ_) Coefficient	β_σVD_	-0.32±0.06		-0.18±0.07	
	β_σODF_	0.15±0.05		0.11±0.05	
	β_σVD⋅ODF_	-0.086±0.02		-0.086±0.02	

Interaction model coefficients ±standard error (SE) and p-values calculated from of the linear mixed effects model with interaction of the predictors estimating logHRW_a_. Distance along the vessel (V_dist_) in disc diameter and ophthalmodynamometric force (ODF) in grams force (g). I_0_ = model intercept, C_VD_ = coefficient of V_dist_,C_ODF_ = coefficient of ODF and C_VD*ODF_ = coefficient of the interaction term V_dist_:ODF. Multivariate coefficient effect size is calculated for the terms in the interaction model using standardized Beta coefficients (β_σn_) expressed in units of standard deviation, where β_σVD_, β_σODF_ and β_σVD⋅ODF_ are the effect sizes for C_VD_, C_ODF_ and C_VD*ODF_ respectively. Conditional R^2^ = Total explanatory power, Marginal R^2^ = Predictor explanatory power.

*** = p<0.00001,

** = p<0.002,

* = p<0.05.

**Fig 9:**
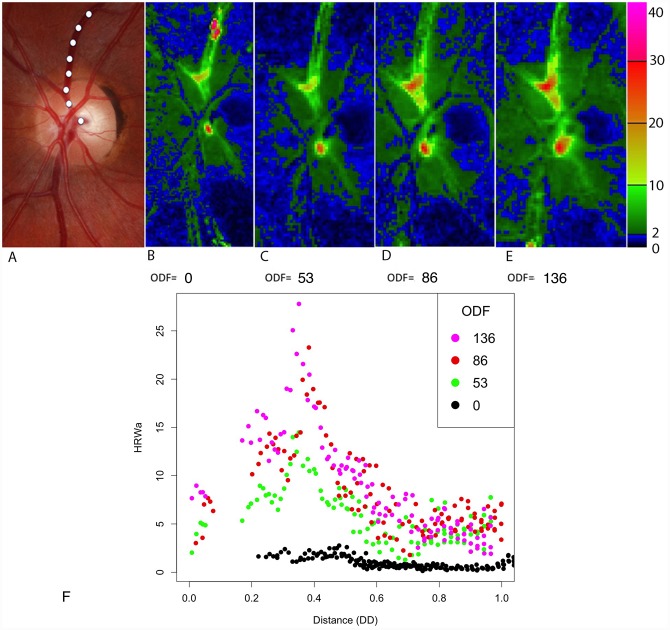
Composite image from a single subject. The distribution of the harmonic regression waveform amplitude (HRW_a_) along the retinal vascular tree for three consecutive cardiac cycles at four ODF values (grams force). (A) Color fundus photograph highlighting the vessel from which graph (F) is compiled for the superior temporal retinal vein as indicated by the white dotted line. (B-E) Heat map representation. Note the increase in the HRW_a_ with increasing ophthalmodynamometric force and the decay as a function of distance in the retinal veins. (F) Scatterplot of the superior retinal vein demonstrating a constant the point of maximum pulsation, in this case 0.38 disc diameter from the optic disc. DD = Disc diameter.

**Fig 10:**
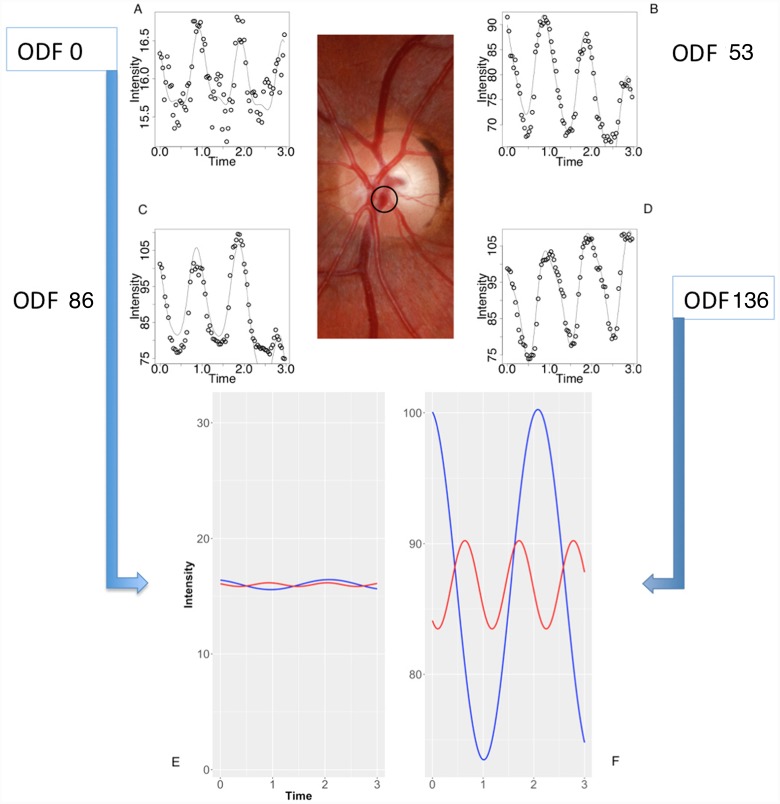
Composite image from the subject from Fig 9. (A-D) The retinal venous pulse harmonic regression waveforms are represented graphically for ophthalmodynamometric force (ODF) 0, 53, 86 and 136 grams force. (E-F) The decomposed first (blue line) and second (red line) Fourier harmonic waves at ODF = 0 at (E) and ODF = 136 grams force at (F) calculated from the Fourier series equation for a single point as highlighted in the central color fundus image. Noted were the increase in the amplitude of the waveforms and the increase in the y-intercept of the regression line with increasing ODF.

**Fig 11:**
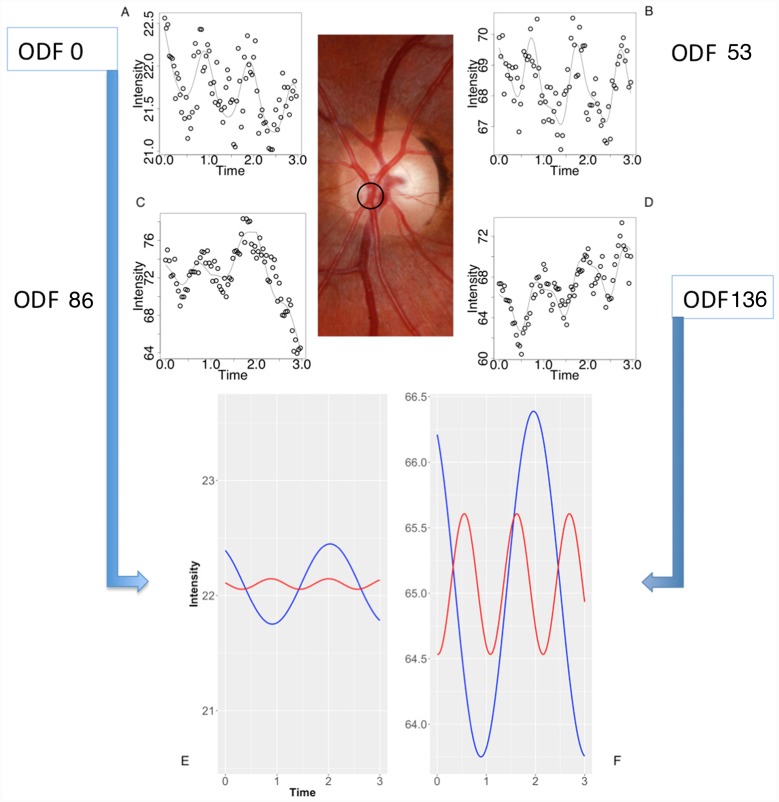
Composite image from the subject from Fig 9. (A-D) The retinal arterial pulse harmonic regression waveforms are represented graphically for ophthalmodynamometric force (ODF) 0, 53, 86 and 136 grams force. (E-F) The decomposed first (blue line) and second (red line) Fourier harmonic waves at ODF = 0 at (E) and ODF = 136 grams force at (F) calculated from the Fourier series equation for a single point as highlighted in the central color fundus image. Noted were the increase in the amplitude of the waveforms and the increase in the y-intercept of the regression line with increasing ODF.

The influence of ODF on the logHRW_a_ spatial distribution profile in the vessel wall and the point of maximum pulsation (logHRW_amax_) is demonstrated in a single vascular segment from a test subject in Fig 9, also demonstrated graphically from a single point in the retinal veins and arteries in Figs 10 and 11 respectively. From the total of 36,619 data points, logHRW_amax_ for each vascular segment (n_total_ = 274, n_arterial_ = 138, n_venous_ = 136) was analyzed at different ODF values, a correlation between the distance of shift in logHRW_amax_ measured from the center of the optic disc with changing ODF failed to achieve statistical significance (p = 0.08, 0.9) for both the venous and arterial systems respectively.

### The Fourier series coefficients (a_n_ and b_n_) for the first and second harmonics

**Fig 12:**
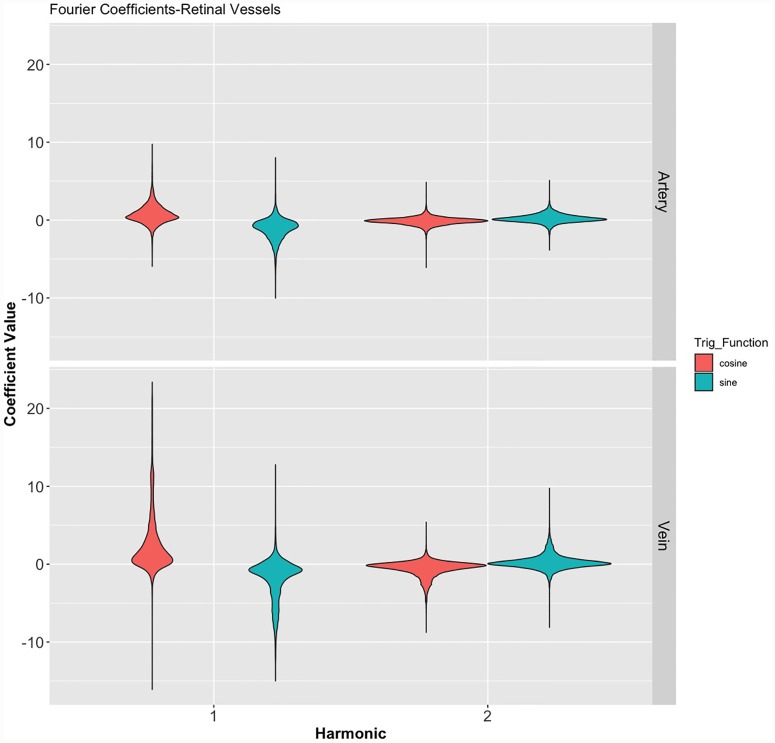
Violin plot of the distribution of the Fourier coefficients of the first and second Fourier harmonics in both vascular systems classified by trigonometric function. Demonstrating the wider range for both the cosine (a_n_) and sine (b_n_) coefficients in the veins, a narrower dispersion of values for the second Fourier harmonics and the disparate distribution, with displacement in the opposite direction across the axis of reference between the a_n_ and b_n_ coefficients in both vascular systems. Numeric values of the figure are outlined in Table 2.

The merits of the coefficient sub-analysis are apparent from Eq 6, as the aim of the study was to investigate amplitude distribution of the HRW in the retinal vascular system, the sub-analysis aimed at investigating if a particular trigonometric wave subset had a favorable correlation with both V_Dist_ and ODF. Table 2 summarizes the numerical statistical properties of the coefficients for the first and second harmonics, which are summarized graphically in Fig 12. In addition to the opposed mean coefficient values of a_n_ and b_n_ within the individual harmonics seen in this figure, a disproportionate contribution from the a_n1_ coefficient to the first Fourier harmonic was noted in the retinal venous system.

**Table 2:** Dispersion and central tendency of the coefficients of the first two harmonics of the retinal arteries and veins.

	Range	Median	IQR	Skewness	Kurtosis
Retinal Veins					
a_n1_	-16.08 to 23.38	0.93	2.52	1.97	4.79
b_n1_	-14.99 to 12.76	-0.81	1.89	-1.16	1.62
a_n2_	-8.75 to 5.39	-0.24	0.82	-1.40	3.96
b_n2_	-8.10 to 9.74	0.09	0.72	0.14	5.51
Retinal Arteries					
a_n1_	-5.93 to 9.73	0.52	1.24	0.94	3.61
b_n1_	-10.02 to 8.01	-0.75	1.38	-0.61	2.71
a_n2_	-6.08 to 4.85	-0.11	0.54	-0.20	7.71
b_n2_	-3.85 to 5.09	0.11	0.56	1.01	3.58

There is a higher median pulsation amplitude, skewness and wider range of the coefficients of the retinal veins compared to the retinal arteries. These findings are demonstrated graphically in Fig 12. IQR = interquartile range, a_n1,2_ = cosine coefficients and b_n1,2_ = sine coefficients of the first and second Fourier harmonics respectively.

The results of the correlation between the cosine (a_n_) and sine (b_n_) components of the trigonometric series with the predictors V_dist_ and ODF are summarized in Table 3, which demonstrated a statistical significance for the correlation with V_dist_ was achieved for all coefficients except the b_n2_ coefficient, in contrast to ODF which failed to achieve statistical significance with all but the a_n1_ coefficient. Therefore, among the coefficients there exists varying correlation with the predictors, with the a_n1_ coefficient the strongest and the b_n2_ the weakest linear correlation for both predictors in both retinal vascular systems.

**Table 3:** Linear mixed effects model of the individual coefficients of the Fourier trigonometric series from the first and second harmonics.

	Coefficient Distance	95% CI	p-value	Coefficient ODF	95% CI	p-value
Retinal Veins						
a_n1_	-3.79±0.78	-5.320 to -2.268	0.000001	0.026±0.0099	0.006 to 0.045	0.01
b_n1_	1.98±0.48	1.033 to 2.93	0.00004	0.00083±0.003	-0.005 to 0.007	0.8
a_n2_	0.498±0.13	0.249 to 0.747	0.0001	-0.005±0.003	-0.01 to 0.0009	0.1
b_n2_	-0.0037±0.17	-0.337 to 0.331	1	-0.004±0.002	-0.008 to 0.0009	0.1
Retinal Arteries						
a_n1_	-1.27±0.37	-1.992 to -0.549	0.0006	0.009±0.04	0.002 to 0.02	0.01
b_n1_	1.026±0.22	0.591 to 1.463	0.000004	-0.0002±0.002	-0.004 to 0.004	0.93
a_n2_	0.21±0.076	0.047 to 0.348	0.01	-0.002±0.002	-0.005 to 0.00091	0.2
b_n2_	-0.041±0.16	-0.347 to 0.267	0.81	-0.0007±0.001	-0.0032 to 0.0021	0.58

Linear mixed effect model correlating the Fourier coefficients (coefficient value±standard error) with distance along the vessel (V_Dist_) and ophthalmodynamometric force (ODF) in both retinal veins and arteries. Statistical significance was achieved for the correlation with V_Dist_ for all coefficients except the b_n2_ coefficient. In contrast ODF failed to achieve statistical significance with a (*p* < 0.05) limit with all but the a_n1_ coefficient for both vascular systems. a_n1,2_ = cosine coefficients and b_n1,2_ = sine coefficients of the first and second Fourier harmonics respectively. 95% CI = 95% confidence interval.

## Discussion

Using mPPG, we described the HRW_a_ distribution and attenuation characteristics of its pulsatile component in both retinal vascular systems non-invasively. The median HRW_a_ was found to be higher in the retinal veins than in the retinal arteries. This is consistent with clinical observations of the retinal venous pulse being visible compared to the arterial pulse in the physiological state [6, 33]. Although the spontaneous venous pulse has been classically described as a binary clinical sign (either present or absent) [6], we demonstrated that modified photo-plethysmography can potentially provide a predictable and quantifiable measurement of the pulse amplitude attenuation. From our previous work, we compared subjective retinal venous pulsation detection with mPPG, the minimum detectable threshold was found to be 5 units (95% confidence interval 4.3 to 6.0) and the area under the receiver operator curve (AU-ROC) was 0.89, which indicated a high accuracy in the detection of pulsatile vascular segments [53]. The reproducibility of this technique demonstrated a coefficient of variation of 13% for vessel pulsation amplitude and 4% for pulsation timing [48]. We are unaware of any other studies in the literature to date that have provided this comparison.

Although it is difficult to detect retinal arterial pulsations clinically, with mPPG we were able to detect the attenuation of the arterial pulse wave at low ODF values and, although the amplification with ODF did not achieve statistical significance (Fig 6), a strong negative linear correlation was detected with V_Dist_. When this attenuation was compared between both vascular systems, we found the attenuation of the pulse wave in the venous system was almost twice that of the arterial attenuation. Several factors contribute to this phenomenon: vessel wall pulse wave attenuation occurs as a consequence of blood viscosity, the transmission of energy to tissues surrounding the blood vessel, pressure wave reflection particularly at the microvascular bed and most significantly the viscoelasticity of the vessel walls [67–70]. The original work demonstrating the significance of vessel wall viscoelasticity on pulse wave attenuation was derived from experimental and theoretical models of pulse wave propagation [71–73]. The first prediction of attenuation of propagated waves was made by Bergel from the measurement of complex elastic properties of excised vessels [74, 75]. This was followed by a series of theoretical and experimental in vivo and in vitro studies, which mainly compared the linearized and non-linearized equations in the prediction of the propagation constant, which allowed attenuation to be expressed in terms of percentage transmission through the systemic vessels as outlined in Table 4.

**Table 4:** Summary of studies in vascular flow related pulse wave attenuation.

Author	Year	Method	Site	Transmission Range (%)	Frequency Range(Hz)
Womersley	1957 [71]	Theoretical	C	C = 0.99-0.92	C = 2-15
			F	F = 1.0-0.96	F = 2-13
Bergel	1961 [76]	In vitro	A	A = 0.98-0.86	2.5-18
			T	T = 0.97-0.72	2.5-18
			F	F = 0.97-0.84	2.5-18
			C	C = 0.97-0.84	2.5-18
Anliker	1968 [77]	In vivo	T	T = 0.7-0.1*	1-3
Mc Donald	1968 [78]	In vitro	C	C = 0.87-0.75	3-14
Mc Donald	1968 [79]	In vivo	C	C = 0.90-0.81	3-11
Mc Donald	1968 [73]	In vitro	C	C = 0.84-0.60	3-11
Wetterer	1968 [75]	In vivo	C	C = 0.98-0.92	3-15
Li	1981 [80]	In vivo	A, F, C, I	C = 0.89-0.47	1-14
Milnor	1975 [25]	In vivo	F	F = 0.85-0.53	3-12
Milnor	1978 [81]	In vivo	F	F = 0.89-0.5	1-13

Arterial flow wave attenuation using various published models and differing methodologies. Transmission was measured as a function of wave frequency/10 cm vessel length. A = Abdominal aorta, C = Carotid, F = Femoral, T = Thoracic aorta, I = Iliac. The theoretical method was calculated using Womersley’s Theory.

* Transmission measured over a 20cm arterial segment. Adapted from McDonald’s blood flow in arteries: theoretical, experimental and clinical principles [75].

A discrepancy between the propagation attenuation coefficient (α) predicted by the theoretical and the experimental models, inexplicable by that derived from the viscosity of blood alone, lead to the conclusion by McDonald et al. of the contribution of vessel wall viscoelasticity being the major factor accounting for pulse wave attenuation [72, 73]. This was demonstrated in subsequent studies, particularly in those studies that analyzed the linearized models of pulse propagation attenuation [70]. Classical elasticity theory is based on an assumption that material is both homogenous and isotropic; however, in biological tissues, particularly in the walls of blood vessels, which are neither homogenous nor isotropic, this assumption is violated. Therefore, vascular distensibility (change in cross-sectional area for a change in pressure) and compliance (change in volume for a change in pressure) both have non-linear dimension-pressure responses [20, 82]. In the systemic vessels the difference in the vessel wall structure, accounts for a difference in viscoelastic responses between the arteries and the veins. The vessel wall consists mainly (70%) of water, with the rest comprised of a material that determines elastic properties (collagen, elastin and smooth muscle) [19]. Consequently, measured arterial compliance curves are curvilinear [83] whereas in the veins this relationship is sigmoidal [84], resulting in a difference in systemic venous compliance being 19 to 24 times of the arterial for an identical transmural pressure and cross-sectional area [82, 85–87]. In the retinal microvascular system, this relationship has not been investigated, however, ultrastructural differences in the vessel walls are well recognized, whereas the retinal arteries are devoid of elastin and consist mainly of layers of collagen with smooth muscle layers, the retinal veins being comprised solely of layers of pericytes surrounded by a collagenous layer of perivascular tissue [88]. This difference in structure is likely to result in a difference in the vascular dimension-pressure response and thereupon a discrepancy in the slopes of attenuation of the HRW_a_ between the vessels as seen in Figs 6, 7 and 8.

The ophthalmodynamometer allows a graded compressive force to be applied to the globe, thereby inducing a measured increase in intraocular pressure. This, in turn, steepens the pressure gradient between the intraocular and retro-ocular compartments and leads to an amplification of retinal vascular pulsations [89, 90]. This sequence was detailed from a single subject in Figs 9 to 11. However, the increase in the slope of the regression line (becoming more negative) with increasing ODF as seen in Figs 7 and 8 and the negative values in the interaction terms in Eqs 13 and 14 involve complex hemodynamic mechanisms, which are poorly understood. Theoretical [91] and experimental models [92–94] have attempted to investigate the retinal and retrobulbar hemodynamic changes from induced IOP elevation. Harris et al. using color doppler imaging, reported changes in the central retinal artery (CRA) including reduced peak systolic velocity, end-diastolic velocity and an increase in the resistive index. Using a method combining Doppler sonography with laser interferometry, Findl et al. concluded that a reduction in the choroidal blood flow in response to an induced increase in IOP was the reason behind a measured reduction in fundus pulsations at the macula and optic disc. Neither investigators detected changes in the hemodynamics of the ophthalmic artery [92, 93]. Therefore an increase in compliance in both the retinal arteries and the retinal veins consequent to a reduction in CRA and possibly choroidal blood flow may be a reason behind the steepening of the regression line with increase ODF observed in Figs 7 and 8. These results are influenced by other systemic factors on the IOP/ocular hemodynamic relationship, including arterial blood pressure, cerebrospinal fluid pressure, orbital tissue pressure, the pressure in the cavernous sinus blood flow, auto-regulation and ocular biomechanics [50], which may make our results difficult to generalize to a wider population.

Lam et al. reported a strong relationship between venous pulsation amplitude with increasing venous diameter, decreasing absolute cup margin distance and decreasing tissue depth overlying the vein [90]. In our subjects the location of the point of maximum venous pulsation, therefore, may be determined by these anatomic characteristics. We found a constant location of the point of maximum venous pulsation in the retinal veins with increasing ODF. To interpret this finding, it is important to consider some of the physiologic mechanisms of the spontaneous venous pulse (SVP). In simplest terms, a variation in the pressure gradient as the vein traverses the lamina cribrosa is the cause of the SVP [95]. However, the spatial distribution of the pulse amplitude along the vein involves a more complex relationship best described by a Starling resistor, which is defined as a collapsible tube in which the pressure external to the tube exceeds the outflow pressure. It predicts that pulsation amplitude is highest near the Starling resistor exit and is attenuated upstream towards the inlet region, this model predicts an expansion of the zone and a constancy of the maximal point of deformation in the wall of the resistor with increasing external force (Fig 9A–9D) [13, 96, 97].

From Eq 6 it is noted that the HRW is a consequence of the summation of the individual trigonometric sine and the cosine waves. The sub-analysis of these individual trigonometric wave coefficients demonstrated that the a_n1_ coefficient in both vascular systems was the only coefficient in the first two harmonics that showed a statistically significant correlation with both predictors (V_Dist_ and ODF). Additionally, this coefficient demonstrated a wider range compared to the sine coefficient to the first harmonic waveform (Fig 12). The reason underpinning the dominance of this waveform is not testable in our current study; however our hypothesis is that this wave may be in phase with the fundamental harmonic of the CSF pulse wave (CSFPW), which is the primary driver for the retinal venous pulse [15]. The fundamental harmonic of the CSFPW is derived from the systemic arterial pressure pulse and is therefore the dominant contributor to this pulse waveform [98]. Consequently, the amplitude and the contour of the CSFPW are dependent on the radius of the arterioles and the characteristics of the walls of the vessels in the cerebrovascular bed [98]. We hypothesize that these factors are likely to be the dominant contributors to the fundamental harmonic of the retinal vascular pulse waveform and reflect factors that influence this waveform indirectly.

The limitations of our described technique include a relatively small sample size. Whereas the interaction model described <65% of the variance, the predictors (V_dist_, ODF) accounted for <20%, with a further 35% was not accounted for in the linear mixed-effects model. A larger sample size would allow assessment of other factors influencing the variance within the model, particularly age. The limits of the sampling frequency (frame rate), determines the number of harmonics extractable from the original signal, a frame rate of 25 FPS allowed correlations to be made on the first two harmonics only. A higher imaging frame rate would theoretically permit the extraction of higher frequency harmonics from the video sequence. Inaccuracies in measuring the pulse amplitude could arise from differences in tissue biomechanics between individuals; furthermore, although baseline intraocular pressure was measured, this was not factored in the analysis. These parameters would influence the ophthalmodynamometric force applied to some extent. Also, transmural pressure is a significant factor governing and linking pulsatile amplitude to intravascular blood flow, as this factor can only be measured invasively, there was no means for this parameter to be factored in our study.

We modeled the attenuation characteristics for the arteries and the veins independently, therefore our work may provide a benchmark for comparison of attenuation characteristics in retinal vascular disease involving the vessel wall. These include age related atherosclerosis [99], hypertension [100], diabetes [101, 102], the influence of pharmacological agents [23] and cerebrospinal fluid pressure dynamics [103, 104].

## Conclusion

Retinal vascular pulse HRW_a_ attenuation was described using modified photo-plethysmography. The predictable attenuation characteristics in normal subjects suggest that this technique may allow the quantification of retinal vascular compliance and other hemodynamic alterations.

## Supporting information

S1 Data (CSV)
